# Evaluation of macular microvascular density using optical coherence tomography angiography in patients with Posner-Schlossman syndrome

**DOI:** 10.1186/s12886-022-02563-z

**Published:** 2022-08-10

**Authors:** Xiu-Juan Guo, Di Chen, li-Jun Zhou, Shu-Ke Luo, Yan Lu, Jing-Jing Guo

**Affiliations:** 1 Department of Ophthalmology, Affiliated Foshan Hospital, Southern Medical University, Foshan, 528000 People’s Republic of China; 2 State Key Laboratory of Ophthalmology, Zhongshan Ophthalmic Centre, Sun Yat-Sen University, Guangzhou, 510000 People’s Republic of China

**Keywords:** Optical coherence tomography angiography, Macular superficial vessel density, Posner-Schlossman syndrome, Intraocular pressure, Central cornea thickness, Ocular perfusion pressure

## Abstract

**Background:**

Optical coherence tomography angiography (OCTA) is a novel technology that provides a noninvasive, dye-less method to visualize the blood vessels of the retina. In the present study, we investigate macular microvascular density and the correlation of ocular and demographic factors using OCTA in Posner-Schlossman syndrome (PSS) patients.

**Methods:**

This is a prospective observational study. All PSS patients and age- and sex-matched healthy subjects underwent complete ophthalmologic examination, and RE, BCVA, IOP, CCT, AL, CMT, GCIPI, RNFL, C/D ratio were recorded. The whole-image vessel density (wiVD) and whole-image perfusion density (wiPD), three-circle (1 mm central ring, 3 mm inner ring, 6 mm outer ring), and four-quadrant segmental VD and PD were calculated.

**Results:**

Seventeen PSS patients and 17 healthy subjects were enrolled in this study. The mean age was 42.65 ± 11.22 years in PSS patients and 42.71 ± 10.50 years in healthy controls. IOP, CCT, and C/D ratio were higher in PSS-attacked eyes, and BCVA, OPP and RNFL thickness was lower than those in the fellow eyes (*p* < 0.05). BCVA and OPP were improved in the PSS-attacked eyes in intermittent period (*p* < 0.05). The wiVD and wiPD were lower in the PSS-affected eyes than in the fellow eyes and in the control eyes in the PSS-attacked period (*p* < 0.05). All segmental VD and PD was lower in the PSS affected eyes than in the healthy control eyes (*p* < 0.05). In intermittent period, the wiVD and wiPD were lower in the PSS-affected eyes than in the fellow eyes (*p* < 0.05). Age, CCT, and SSI were associated with macular wiVD and wiPD in PSS attacked period. Age and CCT were associated with macular wiVD and wiPD in PSS intermittent period.

**Conclusion:**

Decreased macular superficial VD and PD was found in patients with Posner-Schlossman syndrome in attacked period and in remission. Macular wiVD and wiPD were associated with age, CCT and SSI in PSS patients.

**Supplementary Information:**

The online version contains supplementary material available at 10.1186/s12886-022-02563-z.

## Background

Optical coherence tomography angiography (OCTA) is a novel technology that provides a noninvasive, dye-less method to visualize the blood vessels of the retina [1, 2]. OCTA achieves blood vessel image by using commercially available or custom-research oriented algorithms for analysing the variation in OCT signal caused by moving particles, such as red blood cells [3]. The different algorithms used in OCTA are split-spectrum amplitude decorrelation angiography (SSADA), optical microangiography (OMAG), OCT angiography ratio analysis (OCTARA),speckle variance, and correlation mapping et al. [1, 4–8]. SSADA and OMAG are two common commercially available algorithms embedded in OCTA device.

Studies with OCTA revealed retinal vascular factors reduced in glaucoma and vascular factors have been suggested to play a role in glaucoma development and progression [9, 10]. Retinal vessel density (VD) decreased in primary open angle glaucoma (POAG), primary angle-closure disease (PACD), and other secondary glaucoma, such as pseudoexfoliation syndrome (PEX), using OCTA examination [11–13]. Posner-Schlossman syndrome (PSS), also known as glaucomatocyclitic crisis, one of secondary glaucoma, is clinically characterized by recurrent mild, nongranulomatous anterior uveitis and acute elevated intraocular pressure (IOP) [14, 15]. This disease is considered a benign condition because the clinical manifestation of PSS-affected eyes can be completely recovered in the intermittent period in most cases. However, long-term, recurrent episodes may eventually result in optic neuropathy and visual function loss [16]. The microvasculature in PSS patients is unclear in present. The aim of this prospective observational study was to investigate macular microvascular density and the correlation of ocular and demographic factors with macular microvasculature in PSS patients.

## Methods

### Ethics declaration

This study was approved by the Institutional Review Board at Department of Ophthalmology, Affiliated Foshan Hospital, Southern Medical University (No. KJ2019002). All examinations and management were performed in accordance with the Declaration of Helsinki in 1964 and revised in 2020. All subjects recruited in this study signed informed consent forms.

### Study population

A prospective cross-sectional study was conducted at the Department of Ophthalmology, the Second People’s Hospital of Foshan, between December 2019 and June 2022. PSS was diagnosed according to the following criteria: (1) unilateral and recurrent mild inflammation in the anterior chamber; (2) characteristic hoar and white suet-shaped keratic precipitates (KPs); (3) transient episodes of elevated IOP; (4) open angles without iris synechia; and (5) no posterior inflammation. Exclusion criteria included previous antiviral treatment, intraocular surgery, or penetrating ocular injury. The inclusion criteria for the PSS patients were (1) being older than 18 years old, (2) refractive error (RE) beyond ± 6D of the sphere or ± 3D of the cylinder, (3) presence of significant media opacities (e.g., corneal or opacity cataract), (4) history of ocular surgery, (5) history of retinal diseases (e.g., macular degeneration, diabetic retinopathy, retinal detachment and central serous chorioretinopathy), (6) history of systemic diseases, such as hypertension, diabetes mellitus, coronary artery disease, cerebral vascular accident, migraine and obstructive sleep apnoea syndrome (OSAS), and (7) recent episode with IOP controlled under topical anti-glaucoma and/or anti-inflammation medications history, and these medications discontinued less than 1 month.

The inclusion criteria for the healthy subjects were (1) being over 18 years old, (2) having no ocular disease history, (3) having no history of systemic disease, and (4) having best-corrected visual acuity (BCVA) ≥ 20/20, and (5) RE within ± 6D of the sphere or ± 3D of the cylinder.

### Ocular examinations

All subjects underwent complete ophthalmologic examinations, including refractive measurement, BCVA and IOP measurements with Goldmann applanation tonometry, slit-lamp biomicroscopy, indirect fundus ophthalmoscopy, central cornea thickness (CCT), and axial length (AL) measurement using a Lenstar LS 900 (Haag-Streit, Koeniz, Switzerland) and gonioscopy. Arterial systolic blood pressure (SBP) and diastolic blood pressure (DBP) were recorded. Mean arterial pressure (MAP) was calculated with the equation MAP = 1/3SBP + 2/3DBP. Ocular perfusion pressure (OPP) was calculated with the equation OPP = 2/3(MAP-IOP).

Optical coherence tomography (Cirrus 5000 HD-OCT; Carl Zeiss Meditec, Inc., Dublin, CA) was used to assess central macular thickness (CMT, central 1 mm) and macular ganglion cell-inner plexiform layer thickness (GCIPL). A 512 × 128-μm macular cube was used to assess these two macular structure parameters. Both eyes of PSS patients and one eye of healthy subjects underwent OCTA examination using a Zeiss AngioPlex device (Cirrus HD‐OCT 5000, Zeiss Meditec. Inc. Dublin, CA). This OCT device uses an OMAG algorithms with commercial embedded software for analysis (version, 10.0.0.14618), which utilizes both the intensity and phase information from B scans repeated at the same position to delineate blood vessels [1].

**Fig. 1:**
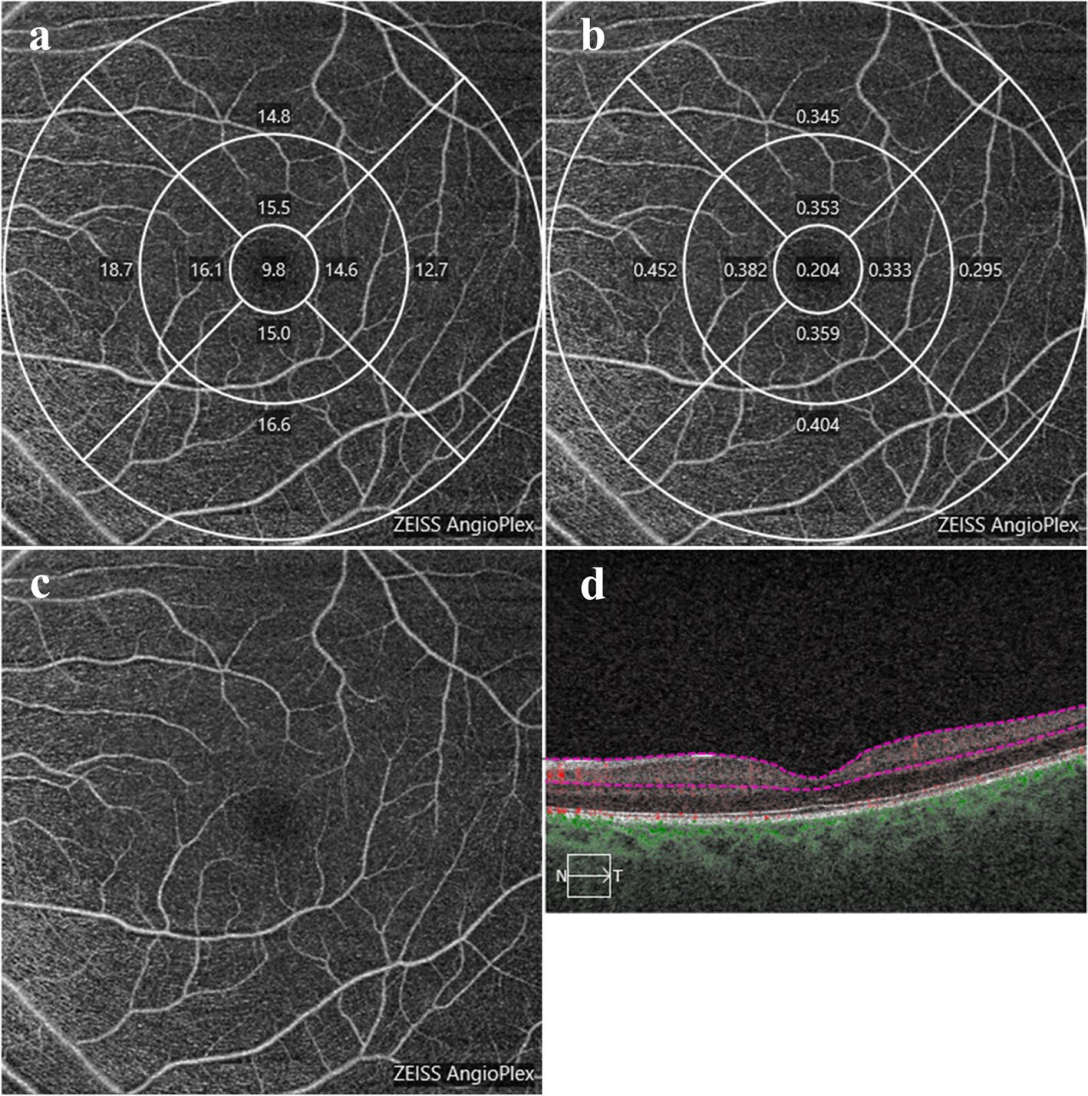
Representation of measurement for macular superficial VD and PD. **a** Three circles of VD (1 mm central ring, 3 mm inner ring, 6 mm outer ring) and four quadrant VD. **b** Three circles of PD (1 mm central ring, 3 mm inner ring, 6 mm outer ring) and four quadrant PD. **c** En face image of superficial capillary plexus. **d** B scan to define superficial capillary plexus (red lines)

Macular miacrovascular denstiy was calculated using a scan rate of 68,000 A-scans per second and with a real-time eye-tracking system (FastTracTM) to reduce motion artefacts. The 6 × 6 mm scan pattern has 350 A-scans in each B-scan along both the horizontal and the vertical directions [3]. The superficial capillary plexus was measured from the internal limiting membrane to the inner plexiform layer [17]. Angiometric software of the Cirrus OCT automatically calculates 2 parameters from the superficial retinal layer slab. VD is defined as the total length of the perfused vasculature per unit area (mm^−1^) in the region of measurement. Perfusion density (PD) is defined as the total area of perfused vasculature (on the binarized image) per unit area in a region of measurement. The OCTA software can automatically calculate the whole-image vessel density (wiVD), three circles of vessel density (1 mm central ring, 3 mm inner ring, 6 mm outer ring), and segmental VD, in which the inner ring and outer ring are further grouped into four quadrants (superior, inferior, nasal and temporal), based on the Early Treatment of Diabetic Retinopathy Study. PD parameters can also be calculated in the same scan (Fig. 1). OCTA images with low image quality, such as low signal strength (less than 7/10), loss of fixation, segmentation error, motion artefacts, and local image loss were excluded.

During the attacked period, the PSS patients were first tested for blood pressure, then general ocular examinations, followed by mydriasis with 0.5% tropicamide, and finally OCT and OCTA examinations were performed. When patients completed all ocular examinations, they were prescribed topical and/or systemically anti-glaucoma medications and topical corticosteroids. Regular follow-up visits were carried out and general ocular examinations were performed. When IOP was under controlled (< 21 mmHg) and KP had disappeared or significantly reduced, the condition was defined as remission [18]. Anti-glaucoma medications and topical corticosteroids were then reduced gradually until the complete withdrawal. When all medications discontinued more than 1 month, we performed second OCT and OCTA examination. In the remission period, in addition to blood pressure and general ocular examinations, visual field was also tested, then mydriasis, and finally OCT and OCTA examinations were performed.

### Statistical analysis

IBM SPSS (SPSS, Chicago, IL, USA) statistical software version 26.0 was used for all statistical analyses. Continuous variables are presented as the mean ± standard deviation. Independent sample t-tests were used to assess the systemic parameters of PSS patients and healthy subjects. Paired t-tests were performed to determine the ocular parameters of PSS-affected eyes and fellow eyes. One-way ANOVA was performed to determine the ocular parameters of all three groups. Categorical variables are expressed as percentages. We performed linear regression analysis to analyse the correlation between macular vascular density and the dependent variables, including systemic and ocular parameters. Variables with *p* < 0.20 in univariate analysis were included in the multivariate analysis. Regression analysis included both eyes of PSS patients in attacked period and in remission, separately. In all statistical analyses, a p value less than 0.05 was considered statistically significant.

## Results

Of the 40 recruited PSS patients, 17 patients were ultimately enrolled in this study. Twenty-three PSS patients were excluded from the analysis. Five patients had recent PSS episodes and their IOP has been under control with topical anti-glaucoma and/or anti-inflammation medications, which were discontinued less than 1 month prior. Three patients had hyper myopia; the refractive error was less than -6D. Two patients had systemic hypertension. Thirteen patients had low-quality OCTA images (including SSI < 7, and or other artefacts).

Thirty-four eyes of 17 PSS patients and 17 eyes of 17 age- and sex-matched healthy subjects were included in this study. The mean age was not significantly different between PSS patients ( 42.65 ± 11.22 years) and healthy controls (42.71 ± 10.50 years). Twelve subjects (70.59%) were male, and 5 subjects (29.41%) were female in these two groups. Blood pressure was not significantly different between healthy subjects (SBP 119.00 ± 11.60 mmHg, DBP 77.06 ± 8.46 mmHg) and PSS patients in acute episode (SBP 115.94 ± 11.42 mmHg, DBP 74.65 ± 6.09 mmHg (*p* > 0.05). Blood pressure was also not significantly different between PSS patients in episode and in their intermittent period (SBP 118.14 ± 8.91 mmHg, DBP 75.41 ± 5.17 mmHg) (*p* > 0.05). The duration of PSS was 21.71 ± 13.36 months. The number of acute episodes in PSS patients was 3.7 ± 2.91.

Table 1 shows the basic ocular parameters. In 17 PSS patients, eleven (64.71%) were affected in the right eye, and 6 (35.29%) were affected in the left eye. One eye in 17 age- and sex-matched normal subjects was included as a control. In episode, no significant differences were found in BCVA, RE, AL, GCIPL, RNFL thickness or CMT between the control eyes, the PSS-affected eyes and the PSS fellow eyes in One-way ANOVA analysis. The IOP and CCT in the normal subjects were less than those in PSS-affected eyes (*p* < 0.001 in IOP and *p* = 0.020 in CCT) and similar to those in the PSS fellow eyes (*p* > 0.05). The C/D ratio in normal subjects were less than those in PSS-affected eyes and in PSS fellow eyes (*p* < 0.001). IOP, CCT, C/D ratio, and OPP were higher in the PSS attacked eyes than those in the fellow eyes (*p* < 0.001 in IOP, *p* = 0.001 in CCT, *p* = 0.008 in C/D, and *p* < 0.001 in OPP). RNFL thickness was thinner in the PSS attacked eyes than in the fellow eyes (*p* = 0.008). Other basic ocular parameters (BCVA, RE, AL, GCIPL, and CMT) were similar in both eyes in attacked period in PSS patients (*P* > 0.05).

**Table 1:** Basic ocular characteristics

	Normal eyes(A)	PSS eyes in attacked period (B)	Fellow eyes in attacked period (C)	PSS eyes in intermittent period (D)	Fellow eyes in intermittent period (E)	p*	p B v/s C	p A v/s B	p A v/s C	P D v/s E	P B v/s D	p C v/s E
BCVA(logMAR)	-0.05 ± 0.09	0.06 ± 0.16	-0.01 ± 0.09	0.01 ± 0.11	-0.03 ± 0.08	0.055	**0.008**	0.050	0.559	0.055	**0.008**	0.083
RE (D)	-0.83 ± 1.72	-0.88 ± 1.55	-0.90 ± 1.90	-0.97 ± 1.95	-0.94 ± 1.88	0.995	0.410	0.941	0.980	0.894	0.508	0.083
IOP (mmHg)	14.64 ± 2.61	38.58 ± 8.56	15.23 ± 4.58	13.64 ± 1.42	14.21 ± 2.43	** < 0.001**	** < 0.001**	** < 0.001**	0.955	0.102	** < 0.001**	0.265
AL (mm)	23.74 ± 1.00	24.24 ± 1.03	24.20 ± 1.20	24.19 ± 1.05	24.21 ± 1.17	0.331	0.587	0.180	0.223	0.868	0.005	0.307
CCT (μm)	542.29 ± 26.62	567.94 ± 36.70	551.24 ± 29.06	545.94 ± 30.45	548.82 ± 31.46	**0.060**	**0.001**	**0.020**	0.406	0.169	** < 0.001**	0.114
GCIPL (um)	85.12 ± 4.64	80.12 ± 12.05	84.65 ± 4.87	85.18 ± 4.81	80.35 ± 7.92	0.141	0.128	0.074	0.864	**0.007**	0.901	0.132
RNFL (um)	95.24 ± 6.92	90.47 ± 14.83	95.71 ± 6.10	87.06 ± 15.02	95.47 ± 5.00	0.133	**0.016**	0.276	0.875	**0.011**	**0.003**	0.821
C/D ratio	0.345 ± 0.10	0.57 ± 0.14	0.48 ± 0.13	0.56 ± 0.14	0.47 ± 0.12	** < 0.001**	**0.008**	** < 0.001**	**0.003**	**0.007**	0.398	0.605
VF (MD)	-	-	-	-1.53 ± 3.76	0.09 ± 0.23	-	-	-	-	0.089	-	-
CMT (um)	247.35 ± 14.50	245.12 ± 15.48	245.35 ± 15.77	249.35 ± 28.01	244.12 ± 15.09	0.896	0.853	0.671	0.704	0.402	0.508	0.220
OPP (mmHg)	50.93 ± 5.22	33.22 ± 9.54	48.79 ± 6.69	50.74 ± 4.41	50.36 ± 4.97	** < 0.001**	** < 0.001**	** < 0.001**	0.401	0.102	** < 0.001**	0.072

Bold values are statistically significant

*BCVA* best-corrected visual acuity, *RE* refractive error, *IOP* intraocular pressure, *AL* axial length, *CCT* central cornea thickness, *GCIPI* ganglion cell-inner plexiform layer thickness, *RNFL* retinal nerve fibre layer, *C/D ratio* cup/disc ratio, *VF (MD)* visual field (mean defect), *CMT* central macular thickness, *OPP* ocular perfusion pressure

p*: One-Way ANOVA was used in three groups (normal eyes, PSS eyes and fellow eyes) in PSS attacked period. p A v/s B and p A v/s C: One-Way ANOVA. p B v/s C, p D v/s E, p B v/s D, and p C v/s E: paired t test

In intermittent period, GCIPL, RNFL, and C/D ratio in PSS attacked eyes were less than those in the fellow eyes (*p* = 0.007 in GCIPL, *P* = 0.011 in RNFL, and *p* = 0.007 in C/D ratio). BCVA and OPP of PSS attacked eyes in intermittent period were improved compared with those in acute period (*p* = 0.008 in BCVA and *p* < 0.001 in OPP). IOP, CCT, and RNFL were decreased in intermittent period than those in attacked period (*p* < 0.001 in IOP and in CCT, *p* = 0.003 in RNFL). No significant differences were found in all basic ocular parameters between attacked period and intermittent period in PSS fellow eyes (*p* > 0.05).

Macular VD parameters are shown in Table 2. In episode, the wiVD was lower in the PSS-affected eyes than in the fellow eyes (*p* = 0.004) and lower than in the control eyes of healthy subjects (*p* < 0.001). There was no significant difference in wiVD between the PSS fellow eyes and the control eyes (*p* = 0.175). In segmental macular VD, except the central ring VD and outer temporal VD, segmental VD was lower in the PSS-affected eyes than in the fellow eyes (*P* < 0.05). All segmental VD was lower in the PSS eyes than in the control eyes (*p* < 0.05). Except for inner temporal VD (*p* = 0.015) and outer temporal VD (*p* = 0.017), segmental VD was similar in the PSS fellow eyes and in the control eyes (*p* > 0.05). There was no significant difference in SSI among the three groups (*p* = 0.167).

**Table 2:** Macular vessel density (VD) parameters (*n* = 17, mean ± SD, p)

	Normal eyes(A)	PSS eyes in attacked period (B)	Fellow eyes in attacked period (C)	PSS eyes in intermittent period (D)	Fellow eyes in intermittent period (E)	p*	p B v/s C	p A v/s B	p A v/s C	P D v/s E	P B v/s D	p C v/s E
wiVD	17.40 ± 2.05	14.16 ± 2.94	16.32 ± 1.72	13.91 ± 3.12	16.06 ± 1.90	**0.001**	**0.004**	** < 0.001**	0.175	**0.007**	0.671	0.233
CR	7.69 ± 3.32	5.71 ± 2.72	6.51 ± 2.45	5.42 ± 2.51	6.65 ± 2.28	0.138	0.319	**0.049**	0.233	0.138	0.512	0.416
IR	17.35 ± 2.40	13.74 ± 3.36	15.69 ± 2.42	13.46 ± 3.21	15.65 ± 2.19	**0.002**	**0.027**	** < 0.001**	0.085	**0.007**	0.715	0.876
OR	17.13 ± 2.70	14.61 ± 2.96	16.78 ± 1.77	14.35 ± 3.12	16.69 ± 1.80	**0.011**	**0.003**	** < 0.001**	0.686	**0.004**	0.665	0.595
IS	17.46 ± 2.85	14.14 ± 3.30	14.16 ± 2.94	13.76 ± 3.92	16.26 ± 2.06	**0.006**	**0.012**	**0.002**	0.154	**0.005**	0.985	0.307
II	17.41 ± 2.04	13.48 ± 3.70	15.75 ± 2.37	13.67 ± 3.09	15.42 ± 2.54	**0.001**	**0.018**	** < 0.001**	0.089	**0.033**	0.775	0.299
IN	16.97 ± 3.21	12.82 ± 4.67	16.11 ± 2.42	13.91 ± 3.35	16.35 ± 2.44	**0.003**	**0.021**	**0.016**	0.761	**0.004**	0.381	0.031
IT	17.63 ± 2.13	13.44 ± 2.92	15.36 ± 2.73	12.52 ± 3.67	15.36 ± 3.05	** < 0.001**	**0.021**	** < 0.001**	**0.015**	**0.010**	0.318	0.985
OS	17.84 ± 2.11	14.59 ± 3.70	16.89 ± 2.16	14.51 ± 3.82	16.78 ± 2.03	**0.004**	**0.005**	**0.013**	0.490	**0.019**	0.899	0.737
OI	17.41 ± 2.06	14.24 ± 2.79	16.85 ± 1.74	14.49 ± 2.68	16.38 ± 2.11	** < 0.001**	**0.001**	** < 0.001**	0.471	**0.014**	0.661	0.134
ON	18.97 ± 2.34	16.95 ± 3.06	19.12 ± 1.00	18.99 ± 1.16	19.18 ± 1.02	**0.014**	**0.005**	**0.014**	0.853	0.197	**0.009**	0.787
OT	16.85 ± 1.99	12.14 ± 4.54	14.06 ± 3.26	11.83 ± 4.40	14.54 ± 3.14	**0.001**	0.104	**0.002**	**0.017**	**0.017**	0.714	0.191
SSI	8.12 ± 0.86	7.94 ± 0.75	8.00 ± 0.94	7.82 ± 0.81	8.06 ± 0.97	0.167	0.805	0.548	0.688	0.104	0.668	0.805

Bold values are statistically significant

*wiVD* whole-image vessel density, *CR* central ring, *IR* inner ring, *OR* outer ring, *IS* inner superior segment, *II* inner inferior segment, *IN* inner nasal segment, *IT* inner temporal segment, *OS* outer superior segment, *OI* outer inferior segment, *ON* outer nasal segment, *OT* outer temporal segment. *SSI* signal strength index

p*: One-Way ANOVA was used in three groups (normal eyes, PSS eyes and fellow eyes) in PSS attacked period. p A v/s B and p A v/s C: One-Way ANOVA. p B v/s C, p D v/s E, p B v/s D, and p C v/s E: paired t test

Except for outer nasal VD (*p* = 0.009), segmental VD was similar in intermittent period and in attacked period in the PSS affected eyes (*p* > 0.05). In intermittent period, except for central ring VD and outer nasal VD, segmental VD was lower in the PSS affected eyes than in the fellow eyes (*p* < 0.05). No significant differences were found in all segmental VD between attacked period and intermittent period in PSS fellow eyes (*p* > 0.05).

Macular PD parameters are shown in Table 3. In attacked period, the wiPD was lower in the PSS-affected eyes than in the fellow eyes (*p* = 0.012) and it was also lower than in the control eyes of healthy subjects (*p* < 0.001). There was no significant difference in wiPD between the PSS fellow eyes and the control eyes (*p* = 0.108). In segmental macular PD, except the central ring PD, inner temporal PD, outer inferior PD, and outer temporal PD, segmental PD was lower in the PSS-affected eyes than in the fellow eyes (*P* < 0.05). All segmental PD was lower in the PSS-affected eyes than in the healthy control eyes (*p* < 0.05). Except for central ring PD (*p* = 0.043), inner temporal PD (*p* = 0.011), and outer temporal PD (*p* = 0.003), segmental PD was similar in the PSS fellow eyes and in the healthy control eyes (*p* > 0.05). Except for outer inferior PD (*p* = 0.046), segmental PD was similar in intermittent period and in attacked period in the PSS-affected eyes (*p* > 0.05).

**Table 3:** Macular perfusion (PD) parameters (*n* = 17, mean ± SD, p)

	Normal eyes(A)	PSS eyes in attacked period (B)	Fellow eyes in attacked period (C)	PSS eyes in intermittent period (D)	Fellow eyes in intermittent period (E)	p*	p B v/s C	p A v/s B	p A v/s C	P D v/s E	P B v/s D	p C v/s E
wiPD	0.426 ± 0.054	0.340 ± 0.082	0.391 ± 0.045	0.330 ± 0.076	0.388 ± 0.051	**0.001**	**0.012**	** < 0.001**	0.108	**0.003**	0.566	0.665
CR	0.182 ± 0.062	0.117 ± 0.063	0.138 ± 0.059	0.140 ± 0.049	0.135 ± 0.057	**0.011**	0.262	**0.003**	**0.043**	0.763	0.074	0.595
IR	0.418 ± 0.063	0.318 ± 0.088	0.369 ± 0.059	0.321 ± 0.078	0.374 ± 0.056	**0.001**	**0.025**	** < 0.001**	0.050	**0.007**	0.874	0.364
OR	0.439 ± 0.053	0.355 ± 0.084	0.405 ± 0.048	0.341 ± 0.078	0.401 ± 0.049	**0.002**	**0.012**	** < 0.001**	0.132	**0.003**	0.392	0.570
IS	0.419 ± 0.071	0.329 ± 0.092	0.377 ± 0.062	0.325 ± 0.099	0.390 ± 0.055	**0.005**	**0.017**	**0.001**	0.120	**0.003**	0.857	0.081
II	0.419 ± 0.057	0.318 ± 0.096	0.370 ± 0.064	0.329 ± 0.068	0.367 ± 0.082	**0.001**	**0.039**	** < 0.001**	0.061	**0.046**	0.577	0.735
IN	0.402 ± 0.057	0.305 ± 0.100	0.371 ± 0.062	0.333 ± 0.078	0.372 ± 0.051	**0.004**	**0.021**	**0.001**	0.267	**0.027**	0.255	0.905
IT	0.417 ± 0.053	0.319 ± 0.078	0.357 ± 0.065	0.296 ± 0.094	0.367 ± 0.082	** < 0.001**	0.057	** < 0.001**	**0.011**	**0.015**	0.368	0.272
OS	0.445 ± 0.059	0.357 ± 0.101	0.411 ± 0.059	0.344 ± 0.091	0.404 ± 0.055	**0.005**	**0.019**	**0.013**	0.280	**0.010**	0.487	0.483
OI	0.431 ± 0.058	0.398 ± 0.051	0.407 ± 0.049	0.344 ± 0.072	0.396 ± 0.053	**0.002**	0.677	**0.001**	0.286	**0.099**	**0.046**	0.154
ON	0.463 ± 0.062	0.404 ± 0.100	0.459 ± 0.055	0.399 ± 0.086	0.462 ± 0.027	**0.045**	**0.033**	**0.026**	0.881	**0.003**	0.854	0.842
OT	0.415 ± 0.054	0.302 ± 0.091	0.332 ± 0.082	0.276 ± 0.11	0.352 ± 0.094	** < 0.001**	0.166	** < 0.001**	**0.003**	**0.015**	0.210	0.274

Bold values are statistically significant

*wiVD* whole-image vessel density, *CR* central ring, *IR* inner ring, *OR* outer ring, *IS* inner superior segment, *II* inner inferior segment, *IN* inner nasal segment, *IT* inner temporal segment, *OS* outer superior segment, *OI* outer inferior segment, *ON* outer nasal segment, *OT* outer temporal segment

p*: One-Way ANOVA was used in three groups (normal eyes, PSS eyes and fellow eyes) in PSS attacked period. p A v/s B and p A v/s C: One-Way ANOVA. p B v/s C, p D v/s E, p B v/s D, and p C v/s E: paired t test

In intermittent period, the wiPD was lower in the PSS-affected eyes than in the fellow eyes (*p* = 0.003). Except for central ring PD, segmental PD was lower in the PSS affected eyes than in the fellow eyes (*p* < 0.05). No significant differences were found in all segmental PD between in attacked period and in intermittent period in the PSS fellow eyes (*p* > 0.05).

In a univariate linear regression analysis, we found that age, sex, BCVA, IOP, CCT, and SSI were associated with macular wiVD and wiPD in PSS attacked period (Table 4). We included factors with p values less than 0.20, which warrants additional motivation to conduct a multivariate linear regression analysis. In multivariate regression analysis, after age and sex controlling, IOP and SSI were associated with wiVD, and only SSI was associated with wiPD in the PSS attacked period (*p* < 0.05) (Table 4).

**Table 4:** Association between macular microvascular density and the dependent variables in episode in the PSS patients

Variables	Macular VD				Macular PD			
	Univariate analysis		Multivariate analysis		Univariate analysis		Multivariate analysis	
	Coefficient (95% CI)	p	Coefficient (95% CI)	p	Coefficient (95% CI)	p	Coefficient (95% CI)	p
Age	-0.075 (-0.156, 0.005)	**0.067**	-0.077 (-0.145, -0.008)	**0.029**	-0.002 (-0.004, 0.000)	**0.050**	-0.002 (-0.004,0.000)	**0.032**
Sex	-1.787 (-3.715, 0.141)	**0.068**	-2.174 (-3.655, -0.692)	**0.006**	-0.049 (-0.101, 0.002)	**0.061**	-0.059 (-0.102,0.016)	**0.009**
OPP	0.051 (-0.030, 0.132)	0.212			0.001 (-0.001, 0.003)	0.342		
BCVA	-5.647 (-12.530, 1.236)	**0.104**	-5.140 (-11.141, 0.860)	0.090	-0.145 (-0.330, 0.040)	**0.121**	-0.129 (-0.303, 0.045)	0.140
RE	0.020 (-0.505, 0.545)	0.938			0.000 (-0.014, 0.014)	0.961		
IOP	-0.056 (-0.122, 0.009)	**0.090**	-0.059 (-0.113, -0.006)	**0.031**	-0.001 (-0.003, 0.001)	**0.161**	-0.001 (-0.003, 0.000)	0.080
AL	0.151 (-0.701, 1.004)	0.720			0.006 (-0.017, 0.029)	0.611		
CCT	-0.031 (-0.057, -0.005)	**0.019**	-0.016 (-0.039, 0.007)	0.170	-0.001 (-0.001, 0.000)	**0.030**	0.000 (-0.001, 0.000)	0.208
GCIPL	-0.024 (-0.125, 0.076)	0.623			-0.001 (-0.004, 0.002)	0.412		
RNFL	-0.004 (-0.086, 0.078)	0.919			0.000 (-0.002, 0.002)	0.792		
C/D	-2.450 (-9.054, 4.154)	0.455			-0.050 (-0.228, 0.128)	0.571		
CMT	0.012 (-0.049, 0.072)	0.703			0.000 (-0.001, 0.002)	0.658		
SSI	1.489 (0.371, 2.607)	**0.011**	-2.174 (-3.655, -0.692)	**0.006**	0.035 (0.004, 0.066)	**0.028**	0.029 (0.003, 0.054)	**0.028**

Bold values are p < 0.2 or statistically significant(*p* < 0.05)

*OPP* ocular perfusion pressure, *BCVA* best-corrected visual acuity, *RE* refractive error, *IOP* intraocular pressure, *AL* axial length, *CCT* central cornea thickness, *GCIPI* ganglion cell-inner plexiform layer thickness, *RNFL* retinal nerve fibre layer, *C/D* ratio cup/disc ratio, *CMT* central macular thickness, SSI signal strength index

In intermittent period, age, CCT, RNFL, C/D, VF, and SSI were associated with macular wiVD and wiPD (Table 5). However, in multivariate regression analysis, after age controlling, only CCT was associated with macular wiVD and wiPD (Table 5).

**Table 5:** Association between macular microvascular density and the dependent variables in remission in the PSS patients

Variables	Macular VD				Macular PD			
	Univariate analysis		Multivariate analysis		Univariate analysis		Multivariate analysis	
	Coefficient (95% CI)	p	Coefficient (95% CI)	p	Coefficient (95% CI)	p	Coefficient (95% CI)	p
Age	-0.088 (-0.172, -0.004)	**0.041**	-0.078 (-0.157,0.001)	0.053	-0.002 (-0.004, 0.000)	**0.031**	-0.002 (-0.004, 0.000)	**0.038**
Sex	-0.008 (-2.162, 2.147)	0.994			0.002 (-0.052, 0.057)	0.933		
OPP	0.002 (-0.004, 0.007)	0.518			0.002 ( -0.004, 0.007)	0.518		
BCVA	-6.045 (-16.004, 3.915)	0.225			-0.155 (-0.408, 0.097)	0.220		
RE	-0.114 (-0.641, 0.412)	0.661			-0.002 ( -0.016, 0.011)	0.748		
IOP	-0.023 (-0.527, 0.481)	0.926			0.001 (-0.012, 0.014)	0.904		
AL	0.083 (-0.825, 0.991)	0.854			0.001 (-0.022, 0.024)	0.929		
CCT	-0.035 (-0.065, -0.005)	**0.026**	-0.040 (-0.068, -0.012)	**0.007**	-0.001 (-0.002, 0.000)	**0.019**	-0.001 (-0.002, 0.000)	**0.006**
GCIPL	0.072 (-0.070, 0.214)	0.312			0.002 (-0.002, 0.005)	0.346		
RNFL	0.065 (-0.016, 0.146)	**0.112**	0.069 (-0.047, 0.186)	0.234	0.001 (-0.001, 0.003)	**0.183**	0.001 (-0.002, 0.004)	0.386
C/D	-5.505 (-12.450, 1.440)	**0.116**	-1.738 (-8.658, 5.183)	0.611	-0.141 (-0.317, 0.035)	**0.112**	-0.048 (-0.219, 0.123)	0.571
VF	0.250 (-0.101, 0.601)	**0.156**	0.010 (-0.469, 0.489)	0.697	0.006 (-0.003, 0.015)	**0.181**	0.001 (-0.011,0.013)	0.882
CMT	-0.014 (-0.058, 0.031)	0.533			0.000 (-0.002, 0.001)	0.373		
SSI	1.226 (0.318, 2.353)	**0.012**	0.665 (-0.389, 1.719)	0.206	0.038 (0.013, 0.063)	**0.005**	0.020 (-0.006,0.046)	0.120

Bold values are p < 0.2 or statistically significant(p < 0.05)

*OPP* ocular perfusion pressure, *BCVA* best-corrected visual acuity, *RE* refractive error, *IOP* intraocular pressure, *AL* axial length, *CCT* central cornea thickness, *GCIPI* ganglion cell-inner plexiform layer thickness, *RNFL* retinal nerve fibre layer, *C/D ratio* cup/disc ratio, *CMT* central macular thickness, *SSI* signal strength index

## Discussion

In this perspective observational study, we investigated macular VD and PD in PSS patients and the correlation of ocular and demographic factors with macular VD and PD using OCTA. In episode, we observed decreased macular VD and PD in PSS-affected eyes compared with PSS fellow eyes and healthy control eyes. IOP and CCT were higher in PSS-affected eyes, and other ocular parameters, including logMAR BCVA, RE, AL, GCIPL, and CMT, were similar in all eyes of these three groups. In remission, though IOP was under controlled, macular VD and PD in PSS-affected eyes have not recovered. In PSS attacked period, regression analysis revealed that macular wiVD was associated with IOP and SSI. Macular wiPD was only associated with SSI. In intermittent period, macular wiVD and wiPD were associated with CCT.

The mechanism of macular microvascular changes in PSS is unclear. A previous study showed that human retinal perfusion is autoregulated [19]. Elevated IOP results in decreased OPP and may impair retinal vessel autoregulation in glaucomatous pathologic processes. Similar to PSS, acute elevated IOP can occur in primary angle closure (PAC), although the mechanisms leading to elevated IOP are different in these two diseases. In PAC, decreased macular VD was found, but with no structural changes [12]. Another similar condition is acute IOP elevation after laser periphery iridectomy (LPI). Ma et al. found that when the IOP increase was greater than 20 mmHg after LPI, macular VD and peripapillary VD decreased significantly [20]. A negative effect of elevated IOP on retinal microcirculation was also found in an animal model [21]. When IOP decreased after glaucoma surgery, a significant increase in VD in the lamina cribrosa and in the peripapillary retinal nerve fibre layer plexus capillary density was found [22, 23]. Peripapillary and macular VD also significantly increased in individuals with high IOP after medical IOP reduction [24, 25]. These results implied that IOP elevation plays an essential role in retinal microvascular changes in patients with glaucoma. Besides elevated IOP, RNFL thickness thinning and C/D enlarge occurred in PSS-affected eyes in episode and in remission. Consistent with these structure alterations, macular VD and PD reduced not only in attacked period, but also in remission in PSS-affected eyes.

This study found that thicker CCT was a predictor of deceased macular VD and PD in attacked period. It is easier to understand that acute elevated IOP in patients with PSS can result in corneal oedema. Unexpectedly, CCT was associated with macular VD and PD in intermittent period. It is need more investigation to explain the results. SSI was significantly associated with macular VD and PD in univariate and multivariate analyses in PSS attacked period, which was consistent with previous results [26–28]. Attention should be given to the effect of SSI on OCTA measurements. For accurate data analyses, recommended SSI values should be obtained. Cirrus HD-OCT recommends ≥ 6 (signal strength, 0 to 10), and even if SSI ≥ 7, increased peripapillary and macular microvascular density will be obtained under better SSI [4, 29]. In our experience, we found it is very difficult to acquire 10 of SSI in all subjects in Cirrus HD-OCT, even if several repeat scans performed and good cooperation of patients (such as no ocular motion and blink) when scanning. So, statistical analysis in SSI is very important before further analysis in microvascular density. In this study, to reduce the impact of SSI, we only included eyes with SSIs greater than 7/10 based on statistical analysis. The SSIs among groups were not significantly different in present study. Corneal oedema is a common cause of lower SSI in PSS-affected eyes. Some PSS eyes with severe corneal oedema were excluded from this study. This may be a limitation of using OCTA to investigate this disease at the acute stage.

In segmental macular vascular density, we observed no significant difference in central ring VD and central ring PD between the two eyes of PSS patients, but it was lower in the PSS-affected eyes than in the control eyes. A possible reason is that the foveal avascular zone (FAZ) is the main structure in this circle, and slight changes in microvascular density in the central ring may be difficult to find, which is supported by the finding that the FAZ was not affected by glaucoma in the Level study [30]. This finding is also consistent with previous studies, which confirmed that VD changed earlier in the parafoveal and perifoveal areas, especially in the perifoveal region [13, 31]. Temple quadrant VD in the inner ring and outer ring was lower in PSS fellow eyes than in control eyes. This needs further investigation.

Most studies implied that age is an important factor associated with macular VD and PD [28, 32–36]. This was consistent with the results of this study. A previous study implied that a long axial length may predict lower retina VD [28]. This association was not found in this study. No hyper-myopic subjects were included in this study. Most of them had no refractive error, which may explain this condition. Previous studies showed that GCIPL thickness decreased in glaucomatous eyes and was associated with decreased macular VD [37]. In the present study, GCIPL thickness was not different among PSS-affected eyes, PSS fellow eyes, and control eyes. In remission, GCIPL thickness was not different between PSS-affected eyes and PSS fellow eyes. No correlation was found between macular VD, macular PD and GCIPL thickness. This finding is consistent with the study conducted by Richter et al., who concluded that OCTA parameters had stronger associations with functional than structural measures of glaucoma [38]. RNFL and C/D ratio were not associated macular VD and PD in PSS acute stage. However, RNFL, C/D ratio and visual field were associated with macular VD and PD in PSS remission stage in univariate regression analysis. RNFL and C/D ratio appear to deteriorate after the episode in this study. Although multivariate regression analysis excluded the correlation between RNFL, C/D ratio, visual field, and macular VD and PD, due to the small sample size, these results needs careful interpretation. Further additional studies need to be performed to investigate the correlates with macular VD and PD in PSS patients.

There are several limitations in this study. First, there was a small sample size in this study. PSS is a relatively rare disease. We included patients according to strict inclusion and exclusion criteria. This resulted in the exclusion of 23 patients from the analysis. Corneal oedema is a leading cause of exclusion, which causes low image quality and low SSI. Therefore, selection bias may exist in this study. The third limitation is that the study just included healthy subjects and the same PSS patients in remission as control. It is better to include a glaucoma control group (non-PSS patients) with similar structure alteration (such as GCIPL thickness and RNFL) and IOP and compare them with PSS patient. Forth, we measured the superficial layer of the VD and PD in the macula, and the deep layer of the VD and PD was not analysed in this study. Finally, we used commercial embedded software to calculate macular microvascular density in this study. It is better to investigate microvascular density with more than one algorithm in the same OCTA device or in different OCTA devices. Studies with larger sample sizes and longitudes, including macular superficial layer and deep layer vasculature measurement designs, and including non-PSS glaucoma patients as control designs are needed in the future. Applying two or more algorithms or more OCTA devices at the same study can more accurately understand retinal vascular density changes.

In conclusion, this study first found that macular VD and PD decreased in patients with Posner-Schlossman syndrome. IOP and SSI were associated with wiVD, and only SSI was associated with wiPD in PSS attacked period. CCT was associated with macular wiVD and wiPD in intermittent period.

## Supplementary Information

**Additional file 1.** 

## Data Availability

All data generated or analysed during this study are included in this published article [and its supplementary information files].

## Abbreviations

VD: Vessel density
PD: Perfusion density
PSS: Posner-Schlossman syndrome
OCTA: Optical coherence tomography angiography
RE: Refractive error
IOP: Intraocular pressure
CCT: Central cornea thickness
AL: Axial length
CMT: Central macular thickness
GCIPI: Ganglion cell-inner plexiform layer thickness
wiVD: Whole-image vessel density
SSI: Signal strength index
POAG: Primary open angle glaucoma
PACD: Primary angle-closure disease
PEX: Pseudoexfoliation syndrome
OSAS: Obstructive sleep apnoea syndrome
BCVA: Best-corrected visual acuity
SBP: Systolic blood pressure
DBP: Diastolic blood pressure
MAP: Mean arterial pressure
OPP: Ocular perfusion pressure
SSADA: Split-spectrum amplitude decorrelation angiography
OMAG: Optical microangiography
OCTARA: OCT angiography ratio analysis
