# Supplementary material for: Evaluation of macular microvascular density using optical coherence tomography angiography in patients with Posner-Schlossman syndrome
Source: BMC Ophthalmol. 2022 Aug 10;22:339. doi: 10.1186/s12886-022-02563-z (PMC9364523; doi:10.1186/s12886-022-02563-z)
Supplement: Supplementary file 1 — Additional file 1. [file 12886_2022_2563_MOESM1_ESM.pdf]

Group	Age	Sex (1=male)	Eye	SBP (acute stage in PSS group)	DBP (acute stage in PSS group)
Normal control	38.00	1	R	110.00	72.00
Normal control	22.00	2	R	95.00	66.00
Normal control	44.00	1	R	123.00	63.00
Normal control	25.00	1	R	111.00	80.00
Normal control	36.00	1	R	120.00	79.00
Normal control	38.00	2	R	125.00	86.00
Normal control	46.00	1	L	115.00	79.00
Normal control	32.00	2	R	117.00	83.00
Normal control	46.00	1	R	131.00	83.00
Normal control	45.00	1	R	119.00	60.00
Normal control	49.00	1	R	116.00	71.00
Normal control	50.00	1	R	118.00	82.00
Normal control	55.00	2	R	135.00	77.00
Normal control	59.00	2	L	140.00	82.00
Normal control	55.00	1	R	101.00	72.00
Normal control	35.00	1	R	132.00	90.00
Normal control	51.00	1	R	115.00	85.00
PSS Fellow eye	31.00	2	L	140.00	85.00
PSS Fellow eye	56.00	1	R	121.00	78.00
PSS Fellow eye	61.00	2	L	128.00	82.00
PSS Fellow eye	35.00	1	L	115.00	75.00
PSS Fellow eye	54.00	2	R	130.00	79.00
PSS Fellow eye	49.00	1	L	114.00	78.00
PSS Fellow eye	51.00	1	R	124.00	80.00
PSS Fellow eye	25.00	1	L	116.00	78.00
PSS Fellow eye	47.00	1	L	125.00	76.00
PSS Fellow eye	19.00	2	R	95.00	65.00
PSS Fellow eye	47.00	1	R	112.00	80.00
PSS Fellow eye	47.00	1	R	105.00	69.00
PSS Fellow eye	48.00	1	L	112.00	71.00
PSS Fellow eye	44.00	1	L	105.00	69.00
PSS Fellow eye	38.00	1	L	105.00	71.00
PSS Fellow eye	34.00	1	L	120.00	65.00
PSS Fellow eye	39.00	2	L	104.00	68.00
PSS attacked eye	31.00	2	R	140.00	85.00
PSS attacked eye	56.00	1	L	121.00	78.00
PSS attacked eye	61.00	2	R	128.00	82.00
PSS attacked eye	35.00	1	R	115.00	75.00
PSS attacked eye	54.00	2	L	130.00	79.00
PSS attacked eye	49.00	1	R	114.00	78.00
PSS attacked eye	51.00	1	L	124.00	80.00
PSS attacked eye	25.00	1	R	116.00	78.00
PSS attacked eye	47.00	1	R	125.00	76.00
PSS attacked eye	19.00	2	L	95.00	65.00
PSS attacked eye	47.00	1	L	112.00	80.00
PSS attacked eye	47.00	1	L	105.00	69.00
PSS attacked eye	48.00	1	R	112.00	71.00
PSS attacked eye	44.00	1	R	105.00	69.00

PSS attacked eye	38.00	1	R	105.00	71.00
PSS attacked eye	34.00	1	R	120.00	65.00
PSS attacked eye	39.00	2	R	104.00	68.00

OPP (acute stage in PSS group)	BCVA (acute stage in PSS	IOP (acute stage in PSS group)	AL (acute stage in PSS group)	RE (acute stage in PSS group)	CCT (acute stage in PSS group)	GCPIL (acute stage in PSS group)
48.24	0.00	12.30	25.03	-3.13	566.00	88.00
41.78	-0.10	13.00	22.60	-0.25	553.00	81.00
46.00	0.00	14.00	25.27	-3.00	579.00	83.00
49.29	0.00	16.40	23.72	-2.13	521.00	92.00
53.11	0.00	13.00	24.62	-2.25	526.00	88.00
53.73	0.00	18.40	23.90	-2.63	546.00	94.00
50.67	-0.10	15.00	23.82	-1.00	524.00	87.00
49.22	0.00	20.50	23.45	-1.75	585.00	80.00
55.53	0.00	15.70	24.40	-1.25	586.00	89.00
44.64	-0.10	12.70	24.21	-0.75	537.00	78.00
45.53	-0.20	17.70	23.43	0.00	542.00	85.00
52.33	0.00	15.50	22.88	-0.25	501.00	77.00
57.56	0.00	10.00	23.51	-0.25	560.00	83.00
57.09	0.00	15.70	22.63	2.88	515.00	86.00
46.04	-0.20	12.60	22.90	-0.25	527.00	85.00
60.53	-0.20	13.20	25.39	-0.25	547.00	83.00
54.53	-0.20	13.20	21.84	1.25	504.00	88.00
59.62	0.00	13.90	24.75	0.25	569.00	80.00
51.89	0.00	14.50	24.88	-0.75	531.00	80.00
57.89	0.00	10.50	23.56	0.00	578.00	87.00
39.16	0.00	29.60	24.12	0.00	523.00	93.00
56.20	0.10	11.70	23.88	-0.50	534.00	87.00
52.33	0.10	11.50	23.14	0.75	563.00	94.00
54.18	0.00	13.40	24.06	-0.50	487.00	86.00
50.11	0.00	15.50	25.85	-4.00	513.00	78.00
53.49	0.00	12.10	24.09	-1.25	536.00	89.00
36.20	-0.20	20.70	23.05	0.00	559.00	79.00
50.78	0.10	14.50	24.73	-0.50	592.00	84.00
44.93	0.00	13.60	24.66	-1.50	548.00	78.00
47.44	-0.10	13.50	22.98	0.75	544.00	83.00
41.20	0.00	19.20	23.01	0.50	600.00	87.00
43.02	0.00	17.80	25.47	-4.50	563.00	84.00
46.56	0.00	13.50	22.22	1.25	553.00	88.00
44.40	-0.20	13.40	26.93	-5.25	578.00	82.00
47.96	-0.10	31.40	23.90	0.25	613.00	82.00
39.49	0.20	33.10	24.93	-0.75	533.00	78.00
39.42	0.20	38.20	23.83	0.00	601.00	88.00
36.09	0.20	34.20	24.10	0.00	529.00	91.00
46.00	0.30	27.00	23.94	-0.50	518.00	82.00
43.33	0.30	25.00	23.15	0.50	557.00	94.00
36.11	0.00	40.50	24.36	-0.75	513.00	78.00
32.11	0.10	42.50	25.75	-3.75	529.00	75.00
38.49	0.10	34.60	24.72	-1.00	539.00	78.00
13.27	-0.20	55.10	23.14	0.00	602.00	79.00
34.91	0.10	38.30	24.74	-0.75	604.00	88.00
33.33	0.20	31.00	24.71	-1.00	559.00	65.00
31.84	-0.10	36.90	23.26	1.00	559.00	91.00
19.27	0.00	52.10	23.24	0.25	619.00	42.00

21.29	0.00	50.40	25.08	-3.75	607.00	84.00
24.56	0.00	46.50	22.68	1.00	575.00	85.00
27.27	-0.20	39.10	26.63	-5.00	598.00	82.00

RNFL(acute stage in PSS group	C/D (acute stage in PSS group	CMT (acute stage in PSS	SSI (acute stage in PSS group)	Central ring VD (acute stage in	Inner ring VD (acute stage in PSS group)
107	0.53	243.00	9.00	8.70	18.50
94	0.31	241.00	9.00	8.50	18.90
95	0.42	224.00	8.00	10.20	17.10
95	0.34	237.00	7.00	7.30	17.90
112	0.23	269.00	7.00	11.00	18.20
102	0.2	251.00	8.00	6.50	14.80
93	0.22	232.00	9.00	6.80	16.60
85	0.35	260.00	9.00	4.20	13.70
97	0.43	272.00	7.00	8.90	17.80
92	0.26	225.00	8.00	13.10	19.00
92	0.48	263.00	9.00	6.80	19.20
90	0.38	256.00	9.00	8.50	19.40
101	0.23	256.00	8.00	9.80	18.60
92	0.38	242.00	7.00	8.10	18.40
99	0.41	243.00	8.00	0.50	18.10
101	0.39	235.00	9.00	10.80	18.70
106	0.3	256.00	7.00	1.00	10.20
104	0.31	243.00	9.00	9.60	19.50
92	0.39	242.00	8.00	6.10	15.30
95	0.32	224.00	8.00	2.70	12.60
95	0.58	219.00	9.00	6.00	17.40
100	0.48	269.00	7.00	6.00	12.50
103	0.58	251.00	8.00	5.20	17.30
92	0.4	234.00	9.00	8.00	18.90
84	0.42	252.00	7.00	6.40	15.90
94	0.39	272.00	9.00	9.20	17.00
94	0.72	226.00	9.00	9.20	19.20
87	0.7	263.00	8.00	10.20	15.40
90	0.63	241.00	8.00	4.50	16.40
101	0.43	256.00	8.00	4.40	13.00
91	0.51	242.00	7.00	3.00	12.40
99	0.49	264.00	7.00	6.60	16.20
101	0.3	235.00	8.00	9.80	15.30
105	0.45	238.00	7.00	3.70	12.50
110	0.29	241.00	8.00	3.30	12.20
89	0.45	243.00	8.00	3.40	11.70
86	0.6	218.00	9.00	2.00	5.80
86	0.64	226.00	8.00	4.80	15.70
84	0.66	265.00	7.00	4.70	13.40
100	0.61	241.00	7.00	2.20	11.60
97	0.3	236.00	9.00	8.40	18.50
78	0.65	250.00	9.00	7.00	17.30
66	0.61	281.00	8.00	9.60	17.10
99	0.75	232.00	8.00	5.70	14.90
91	0.76	255.00	9.00	10.50	17.20
53	0.72	244.00	8.00	5.60	17.00
98	0.58	253.00	8.00	8.90	14.90
98	0.44	240.00	7.00	9.10	14.00

90	0.61	266.00	7.00	3.10	11.20
101	0.6	236.00	7.00	4.40	9.20
112	0.45	240.00	8.00	4.40	11.80

Outer ring VD (acute stage in PSS group)	WiVD (acute stage in PSS	Inner superior VD (acute stage in PSS group)	Inner temporal VD (acute stage in PSS group)	Inner inferior VD (acute stage in PSS group)
19.30	18.80	18.50	18.30	18.60
9.80	19.30	18.90	19.10	18.50
17.20	17.00	17.50	16.00	16.80
18.90	18.30	18.20	17.60	17.70
19.20	18.70	18.50	17.70	18.10
15.40	15.00	15.40	14.70	13.90
16.30	16.10	17.10	18.70	16.20
15.90	15.10	14.10	17.50	12.80
17.70	17.50	18.00	17.20	17.90
19.00	18.80	19.30	19.90	18.00
19.30	18.90	19.50	19.60	19.10
18.70	18.50	19.10	19.40	20.00
18.50	18.30	18.40	18.90	18.60
18.70	18.30	18.90	18.30	18.00
18.40	17.90	18.00	17.60	19.30
17.20	18.10	19.50	18.10	18.50
11.80	11.20	7.90	11.10	14.00
19.00	18.80	20.40	19.10	18.90
16.40	15.90	16.30	12.90	16.50
15.20	14.30	10.50	12.70	12.90
18.40	17.80	18.00	17.10	16.60
12.50	13.60	15.30	13.20	13.80
17.90	17.40	16.30	17.00	18.00
18.90	18.60	18.70	18.60	19.30
17.60	16.90	16.40	15.90	15.40
18.10	17.60	17.00	16.60	17.00
19.10	18.90	19.40	19.50	18.70
15.80	15.50	15.50	15.50	15.30
16.80	16.40	15.90	17.70	15.90
16.30	15.20	15.40	9.90	12.70
15.90	14.70	12.40	12.90	13.50
17.10	16.60	15.70	15.80	17.10
15.70	15.40	15.50	14.60	15.00
14.60	13.80	13.80	12.20	11.10
14.80	13.90	13.70	13.40	11.30
15.50	14.30	14.30	11.50	11.70
7.00	6.60	6.00	7.80	4.30
17.60	16.80	16.70	13.60	16.10
11.30	11.60	12.60	12.70	12.80
12.50	12.00	10.20	11.40	13.30
18.20	18.20	18.30	18.60	18.70
17.30	16.70	16.40	18.00	16.30
15.90	16.00	17.80	10.80	17.20
15.90	15.40	15.60	15.40	14.30
17.50	17.20	17.40	15.90	17.20
14.60	14.90	16.40	16.60	17.70
16.70	16.10	16.60	14.40	14.00
16.40	15.70	14.00	14.50	13.90

12.60	12.00	11.80	12.80	10.50
11.10	10.50	10.30	9.40	8.70
13.50	12.90	12.20	11.70	11.10

Inner nasal VD (acute stage in PSS group)	Outer superior VD (acute stage in PSS group)	Outer temporal VD (acute stage in PSS group)	Outer inferior VD (acute stage in PSS group)
18.70	19.20	17.80	19.50
19.10	19.60	18.80	19.90
18.10	18.00	14.60	16.70
18.00	19.00	17.80	18.10
18.60	19.20	17.50	19.20
15.30	16.00	15.00	13.50
14.50	16.70	17.70	15.60
10.50	16.30	15.30	14.10
18.10	17.90	16.00	17.30
18.80	18.80	18.40	18.70
18.60	19.30	18.40	19.20
19.00	18.00	18.50	18.60
18.50	18.80	17.40	18.20
18.40	18.20	17.90	18.80
17.60	18.20	17.70	18.30
18.70	19.30	16.70	16.50
7.90	10.80	10.90	13.80
19.90	19.40	17.20	18.60
15.40	17.40	13.40	15.80
12.90	15.90	12.70	15.10
17.90	17.70	16.50	19.10
16.50	10.00	9.50	12.50
18.10	17.10	15.40	19.20
18.80	19.40	18.60	18.40
15.80	17.80	16.70	16.80
17.40	18.20	16.50	17.90
19.40	18.90	18.30	19.00
15.20	16.10	10.90	16.20
16.30	17.10	15.30	16.00
14.10	17.50	9.90	16.10
10.90	16.30	13.90	15.60
16.10	17.20	14.40	17.20
16.10	14.80	12.70	16.60
13.00	16.40	7.20	16.40
1.50	14.90	14.40	13.40
9.30	16.40	14.60	15.00
5.30	5.70	7.60	7.40
16.40	17.90	15.90	17.50
15.50	8.50	7.30	11.40
11.60	12.10	7.10	13.90
18.00	18.40	18.10	17.70
14.00	17.30	17.30	16.80
16.80	17.60	10.80	15.50
14.20	16.70	15.40	14.60
18.20	18.00	14.70	17.40
17.50	12.20	14.00	14.30
14.50	17.90	14.00	16.50
14.40	15.60	14.50	15.70

10.10	11.90	1.00	11.50
8.30	11.40	8.10	11.60
12.30	15.60	11.50	11.80

Outer nasal VD (acute stage in PSS group)	Central ring PD (acute stage in PSS group)	Inner ring PD (acute stage in PSS group)	Outer ring PD (acute stage in PSS group)
20.50	0.192	0.452	0.475
20.70	0.181	0.452	0.485
19.50	0.219	0.404	0.42
20.50	0.165	0.425	0.465
20.50	0.247	0.417	0.46
17.10	0.137	0.343	0.365
15.20	0.142	0.385	0.396
17.90	0.194	0.325	0.392
19.60	0.195	0.492	0.437
19.90	0.306	0.466	0.481
20.30	0.156	0.458	0.478
19.60	0.196	0.465	0.478
19.50	0.205	0.447	0.461
20.00	0.178	0.444	0.46
19.50	0.111	0.44	0.469
20.40	0.246	0.451	0.451
11.80	0.018	0.241	0.284
20.70	0.224	0.47	0.472
19.00	0.129	0.367	0.41
16.90	0.054	0.285	0.364
20.20	0.1	0.368	0.432
17.90	0.11	0.353	0.285
20.00	0.116	0.408	0.436
19.30	0.176	0.448	0.466
19.10	0.131	0.377	0.437
19.60	0.213	0.411	0.392
20.20	0.214	0.452	0.472
19.90	0.216	0.355	0.391
18.80	0.094	0.383	0.41
18.90	0.087	0.298	0.388
17.70	0.062	0.284	0.387
19.60	0.141	0.378	0.424
18.70	0.204	0.357	0.374
18.50	0.075	0.276	0.347
16.40	0.068	0.278	0.349
16.00	0.067	0.285	0.387
7.50	0.023	0.093	0.131
19.20	0.1	0.368	0.432
17.80	0.1	0.303	0.264
17.00	0.041	0.261	0.296
19.70	0.186	0.442	0.455
17.60	0.151	0.381	0.433
19.80	0.213	0.411	0.392
16.70	0.08	0.352	0.407
19.90	0.223	0.412	0.432
17.80	0.111	0.408	0.361
18.50	0.181	0.344	0.405
19.90	0.197	0.33	0.407

15.80	0.063	0.257	0.304
13.40	0.088	0.204	0.26
15.20	0.089	0.269	0.323

WiPD (acute stage in PSS group)	Inner superior PD (acute stage in PSS group)	Inner temporal PD (acute stage in PSS group)	Inner inferior PD (acute stage in PSS group)
0.462	0.457	0.435	0.47
0.469	0.455	0.449	0.45
0.411	0.433	0.372	0.39
0.448	0.435	0.419	0.417
0.444	0.43	0.403	0.405
0.354	0.351	0.339	0.328
0.386	0.396	0.444	0.375
0.369	0.354	0.41	0.289
0.429	0.434	0.411	0.433
0.473	0.468	0.472	0.473
0.465	0.462	0.464	0.464
0.467	0.471	0.458	0.481
0.451	0.441	0.435	0.454
0.448	0.44	0.452	0.449
0.452	0.436	0.425	0.463
0.445	0.472	0.439	0.446
0.267	0.183	0.258	0.34
0.464	0.481	0.449	0.469
0.392	0.399	0.298	0.406
0.338	0.236	0.324	0.29
0.408	0.385	0.371	0.349
0.319	0.368	0.312	0.337
0.421	0.38	0.389	0.433
0.454	0.457	0.441	0.458
0.415	0.404	0.365	0.373
0.391	0.436	0.401	0.4
0.46	0.457	0.451	0.454
0.378	0.356	0.36	0.355
0.395	0.382	0.413	0.366
0.359	0.356	0.221	0.288
0.355	0.279	0.298	0.314
0.406	0.371	0.373	0.402
0.365	0.353	0.333	0.359
0.324	0.316	0.267	0.243
0.326	0.31	0.297	0.261
0.355	0.338	0.283	0.287
0.119	0.092	0.145	0.079
0.408	0.387	0.386	0.387
0.268	0.281	0.287	0.293
0.281	0.227	0.252	0.314
0.445	0.441	0.439	0.456
0.414	0.405	0.419	0.383
0.391	0.436	0.401	0.4
0.385	0.348	0.354	0.36
0.422	0.421	0.369	0.41
0.365	0.393	0.393	0.426
0.385	0.395	0.315	0.332
0.384	0.325	0.313	0.346

0.287	0.276	0.291	0.238
0.243	0.228	0.21	0.192
0.305	0.286	0.263	0.246

Inner nasal PD (acute stage in PSS group)	Outer superior PD (acute stage in PSS group)	Outer temporal PD (acute stage in PSS group)	Outer inferior PD (acute stage in PSS group)
0.446	0.482	0.44	0.467
0.453	0.486	0.461	0.494
0.42	0.45	0.351	0.397
0.427	0.486	0.444	0.442
0.431	0.472	0.416	0.467
0.352	0.384	0.359	0.305
0.326	0.421	0.436	0.375
0.247	0.402	0.376	0.342
0.439	0.453	0.383	0.433
0.451	0.481	0.46	0.472
0.441	0.48	0.457	0.484
0.449	0.474	0.464	0.489
0.458	0.461	0.45	0.456
0.438	0.449	0.439	0.462
0.434	0.467	0.453	0.481
0.448	0.471	0.41	0.408
0.182	0.249	0.263	0.345
0.48	0.482	0.425	0.46
0.366	0.431	0.334	0.395
0.288	0.387	0.304	0.357
0.365	0.421	0.376	0.469
0.395	0.221	0.207	0.283
0.43	0.419	0.372	0.472
0.436	0.469	0.461	0.464
0.367	0.44	0.411	0.414
0.408	0.428	0.262	0.382
0.449	0.472	0.442	0.454
0.35	0.394	0.263	0.406
0.37	0.42	0.369	0.386
0.327	0.429	0.281	0.381
0.243	0.4	0.335	0.377
0.368	0.434	0.349	0.431
0.382	0.345	0.295	0.404
0.278	0.4	0.162	0.382
0.245	0.347	0.341	0.317
0.232	0.408	0.359	0.381
0.055	0.097	0.154	0.156
0.311	0.446	0.379	0.435
0.352	0.211	0.172	0.249
0.251	0.294	0.163	0.333
0.434	0.45	0.445	0.44
0.318	0.437	0.434	0.426
0.408	0.428	0.262	0.382
0.345	0.423	0.335	0.421
0.448	0.45	0.356	0.426
0.42	0.303	0.339	0.355
0.333	0.446	0.329	0.399
0.336	0.391	0.353	0.388

0.224	0.292	0.26	0.283
0.186	0.264	0.191	0.275
0.28	0.386	0.269	0.275

Outer nasal PD (acute stage in PSS group)	PSS duration (months)	Number of episodes (times)	SBP (intermittent stage in PSS group)
0.511			
0.499			
0.484			
0.487			
0.48			
0.414			
0.351			
0.45			
0.481			
0.505			
0.492			
0.486			
0.478			
0.488			
0.478			
0.511			
0.278			
0.52	48	10	135
0.474	12	1	115
0.408	8	1	130
0.487	15	3	115
0.432	7	1	138
0.482	24	3	120
0.468	7	1	123
0.484	36	3	115
0.501	24	4	120
0.485	6	1	110
0.503	36	9	118
0.461	40	8	116
0.457	9	1	118
0.432	18	4	106
0.483	36	5	110
0.452	25	3	116
0.278	18	5	108
0.392	48	10	135
0.399	12	1	115
0.118	8	1	130
0.467	15	3	115
0.426	7	1	138
0.251	24	3	120
0.485	7	1	123
0.436	36	3	115
0.501	24	4	120
0.452	6	1	110
0.496	36	9	118
0.448	40	8	116
0.447	9	1	118
0.495	18	4	106

0.381	36	5	110
0.31	25	3	116
0.363	18	5	108
	13.35928846	2.9104275	

DBP (intermittent stage in PSS group)	OPP (intermittent stage in PSS group)	BCVA (intermittent stage in PSS group)	IOP (intermittent stage in PSS group)
78	57.33	0	11
75	50.02	0	13.3
85	58.47	0	12.3
78	49.29	0	16.4
80	58.02	0	12.3
79	54.91	0.1	10.3
79	53.51	0	13.4
78	49.22	0	16.5
81	53.13	0	14.3
70	43.22	-0.2	18.5
77	48.38	0	18.1
71	47.8	0	14.3
73	50	-0.1	13
65	40.78	0	17.5
73	47.22	0	14.5
68	47	-0.1	13.5
72	47.73	-0.2	12.4
78	56.67	-0.1	12
75	50.62	0.1	12.4
85	57	0	14.5
78	50.16	0	15.1
80	58.02	0.2	12.3
79	54.31	0.2	11.2
79	53.44	0	13.5
78	50.56	0	14.5
81	54.27	0.1	12.6
70	44.69	-0.2	16.3
77	50.11	0.1	15.5
71	48.4	0	13.4
73	50	-0.1	13
65	42.24	0	15.3

73	47.89	0	13.5
68	46.47	0	14.3
72	47.67	-0.2	12.5

AL (intermittent stage in PSS group)	RE (intermittent stage in PSS group)	CCT (intermittent stage in PSS group)	GCPIL (intermittent stage in PSS group)
24.73	0.25	570	80
24.89	-0.75	529	80
23.58	0	574	88
24.18	0	523	92
23.98	-0.5	530	87
23.15	0.5	557	94
24.11	-0.75	480	86
25.82	-4	515	79
24.11	-1.25	525	90
23.03	0	554	81
24.71	-0.5	605	88
24.64	-1.5	545	78
22.96	0.75	548	82
23.05	0.25	601	87
25.43	-4.5	558	87
22.27	1.25	540	88
26.91	-5.25	576	81
23.75	-2	565	79
24.97	-0.5	525	79
23.9	0	573	80
24.03	0	529	87
23.91	-0.5	511	80
23.05	0.5	547	100
24.28	-0.5	478	77
25.65	-4.75	519	72
24.68	-1.25	524	77
23.04	0	555	78
24.59	-1.25	589	84
24.63	1	537	65
23.26	0.75	540	90
23.21	0.25	599	70

25.11	-4	568	81
22.61	1	552	85
26.64	-5.25	570	82

RNFL(intermittent stage in PSS group	C/D(intermittent stage in PSS group	MD	CMT (intermittent stage in PSS group)	SSI (intermittent stage in PSS group)
95	0.3	0.89	246	10
92	0.4	0.12	241	8
94	0.32	-0.15	225	8
97	0.57	0.2	223	10
99	0.47	0.18	265	7
97	0.61	0.13	245	8
93	0.4	0.03	235	8
84	0.43	0.05	251	8
90	0.38	-0.16	275	9
94	0.7	0.08	225	8
97	0.69	0.03	255	7
90	0.62	-0.08	231	7
102	0.45	0.09	252	8
97	0.49	0.1	243	7
96	0.48	-0.03	265	9
101	0.31	0.08	233	7
105	0.45	0.03	240	8
96	0.28	0.08	232	10
87	0.41	-0.06	242	8
80	0.61	-0.12	215	8
82	0.68	0.11	225	9
80	0.7	0.02	263	7
101	0.6	0.06	245	8
96	0.32	-0.05	238	8
71	0.68	-4.36	246	7
63	0.56	-8.23	272	8
99	0.75	-0.06	233	8
88	0.73	0.1	255	7
53	0.68	-13.4	236	7
97	0.56	-0.05	248	8
90	0.49	0.03	343	7

83	0.45	-0.06	263	8
101	0.56	0.02	240	8
113	0.48	0.01	243	7

Central ring VD (intermittent stage in PSS group)	Inner ring VD (intermittent stage in PSS group)	Outer ring VD (intermittent stage in PSS group)	WiVD (intermittent stage in PSS group)	Inner superior VD (intermittent stage in
8.5	19.4	18.8	19.1	19.5
4.8	12.9	14.6	14	16.3
3.5	13	15.2	14	12.5
6.5	16.43	17.78	17.1	18.4
6	16.1	13.6	14.8	16.4
5.5	17	18	17.4	15.3
8.1	18.4	18.9	18.7	18.5
6.2	15.5	17.97	14.9	15.9
8.9	17.2	18.13	17.6	16.8
9.4	19.1	19.3	19.2	19.8
10	14.5	15.8	15.7	16
4.8	16.2	17.2	16.6	14.6
5	13.6	16	14.8	16
3.2	12.3	14.2	13.2	13
8.4	15.9	17.6	16.4	17.5
10	15.4	15.7	15.6	15.8
4.2	13.1	14.9	14	14.1
6.5	17.5	18.7	18.1	18.4
5.6	15.5	16.3	15.8	16.9
2.3	5.9	7.7	6.8	5
5	16.1	17.8	16.9	17.1
2.9	13.9	13.9	13.6	14.8
2.3	11.3	13.3	12.3	9.7
9	18.2	18.6	18.4	17.8
6.8	16.1	16.6	16.6	16.5
9.1	15.7	15.1	15.4	17.8
6	14.8	15.7	15.2	14.9
5.7	10.8	12	11.6	10.6
3.2	11.5	9.6	9.9	13
9	14.7	16.8	15.7	16.6
8.5	10.8	13.1	12.3	10

1.5	9.6	12.2	11.3	8.1
3.8	11.8	11.3	11.5	12.3
5	14.7	15.3	15	14.5

Inner temporal VD (intermittent stage in PSS	Inner inferior VD (intermittent stage in PSS	Inner nasal VD (intermittent stage in PSS	Outer superior VD (intermittent stage in PSS	Outer temporal VD (intermittent stage in PSS
20.4	19	18.8	18.9	17.2
9.1	12.3	14.1	16.1	9.6
13	13	13.5	16.1	13
16.9	15.8	14.6	18.1	15.8
14.5	15.2	15.9	13.5	10.1
18.1	17.5	17.1	16.8	16.3
18.9	18.7	17.8	19.3	19.2
16.3	15.4	14.9	16.9	17.8
16.8	17.2	18	19.2	16.5
18.9	18.9	18.8	19.2	19
16	15.5	15.3	14.5	12
16.8	16.5	17	17.1	17
10.2	13.4	14.8	16.9	12.1
14	11.2	11	12.4	15
14.2	16.5	15.5	18.3	13.3
14	15	16.8	15	13.5
13	11	14.2	16.8	9.8
17.8	17.2	16.7	19.1	18.4
13.2	14.9	17.2	17.9	12.1
8.1	5.1	5.6	6.1	8
14.5	15.9	16.7	18.1	16.2
11.9	12.9	15.7	14.5	8.9
11.4	12.3	12.1	13.5	7.9
18.5	18.7	17.9	18.4	17.9
17.2	16.3	14.2	17.3	16.8
11.2	16.8	16.8	17.2	9.8
15.6	13.9	14.8	15.7	16.7
9	11.8	11.9	12.6	6.9
7.2	12	13.9	9	5.8
13.8	13.9	14.5	16.9	15.2
10.5	14.3	8.6	13.5	13.3

6.8	13.1	10.3	10.4	5.8
10.5	11	13.2	9.8	9
15.7	12.3	16.4	16.7	12.4

Outer inferior VD (intermittent stage in PSS	Outer nasal VD (intermittent stage in PSS group)	Central ring PD (intermittent stage in PSS	Inner ring PD (intermittent stage in PSS group)	Outer ring PD (intermittent stage in PSS group)
19.9	19.4	0.191	0.476	0.458
14.4	18.3	0.103	0.301	0.343
14.6	19.3	0.08	0.303	0.36
17.2	20	0.089	0.38	0.42
13.2	18.5	0.157	0.371	0.33
19.3	19.9	0.126	0.405	0.435
17.5	19.7	0.176	0.45	0.461
17	20.2	0.11	0.371	0.421
16.8	20	0.196	0.412	0.46
19.1	20.1	0.201	0.46	0.477
16.7	20	0.218	0.376	0.384
17.1	17.9	0.071	0.388	0.399
15.6	19.5	0.126	0.317	0.41
13.3	16.3	0.048	0.29	0.335
18.1	18.5	0.132	0.37	0.412
15.1	19.2	0.218	0.371	0.378
13.6	19.3	0.052	0.311	0.33
18.4	19.2	0.156	0.445	0.458
15.3	19.8	0.139	0.371	0.389
8.5	8.4	0.12	0.168	0.192
17.8	18.9	0.156	0.382	0.421
12.7	19.5	0.11	0.325	0.329
13.8	18	0.068	0.257	0.316
18.1	20	0.196	0.422	0.453
16.8	17.9	0.142	0.382	0.411
14.5	18.9	0.198	0.373	0.354
13.5	16.8	0.137	0.363	0.369
13	15.4	0.136	0.249	0.279
10.7	13	0.09	0.269	0.214
17	17.9	0.196	0.36	0.394
14.1	10.8	0.193	0.248	0.311

14.7	19.2	0.02	0.215	0.299
12.1	14.3	0.153	0.275	0.257
15.3	16.8	0.176	0.349	0.344
	16.75294118			

WiPD (intermittent stage in PSS group)	Inner superior PD (intermittent stage in PSS	Inner temporal PD (intermittent stage in PSS	Inner inferior PD (intermittent stage in PSS	Inner nasal PD (intermittent stage in PSS group)
0.467	0.482	0.506	0.465	0.453
0.322	0.383	0.197	0.286	0.341
0.34	0.292	0.303	0.298	0.321
0.42	0.448	0.406	0.38	0.305
0.34	0.391	0.349	0.363	0.383
0.419	0.361	0.441	0.416	0.402
0.456	0.453	0.459	0.456	0.425
0.394	0.382	0.384	0.363	0.358
0.436	0.406	0.405	0.406	0.432
0.474	0.486	0.458	0.46	0.421
0.379	0.381	0.382	0.372	0.369
0.393	0.355	0.404	0.395	0.401
0.366	0.382	0.221	0.311	0.356
0.312	0.299	0.341	0.259	0.261
0.39	0.415	0.342	0.395	0.343
0.37	0.381	0.34	0.361	0.405
0.32	0.341	0.306	0.258	0.342
0.45	0.457	0.453	0.45	0.421
0.379	0.4	0.322	0.359	0.403
0.18	0.128	0.176	0.192	0.176
0.403	0.404	0.352	0.383	0.39
0.326	0.356	0.279	0.291	0.374
0.285	0.206	0.259	0.283	0.28
0.437	0.428	0.429	0.426	0.403
0.396	0.394	0.407	0.386	0.342
0.363	0.431	0.259	0.403	0.402
0.365	0.356	0.372	0.338	0.389
0.264	0.246	0.21	0.271	0.272
0.242	0.296	0.163	0.281	0.339
0.377	0.394	0.348	0.349	0.352
0.279	0.22	0.232	0.344	0.196

0.257	0.179	0.158	0.302	0.221
0.266	0.285	0.243	0.259	0.313
0.346	0.349	0.376	0.283	0.388

Outer superior PD (intermittent stage in PSS	Outer temporal PD (intermittent stage in PSS	Outer inferior PD (intermittent stage in PSS	Outer nasal PD (intermittent stage in PSS group)
0.469	0.406	0.483	0.478
0.38	0.201	0.345	0.446
0.382	0.301	0.353	0.456
0.44	0.365	0.406	0.486
0.311	0.282	0.302	0.453
0.401	0.383	0.473	0.486
0.474	0.473	0.423	0.484
0.407	0.426	0.41	0.462
0.471	0.48	0.403	0.489
0.472	0.471	0.472	0.493
0.352	0.296	0.401	0.49
0.403	0.399	0.404	0.432
0.408	0.405	0.378	0.463
0.288	0.357	0.323	0.386
0.445	0.314	0.442	0.449
0.358	0.287	0.373	0.45
0.405	0.143	0.346	0.445
0.467	0.447	0.451	0.468
0.431	0.281	0.362	0.482
0.189	0.188	0.196	0.195
0.441	0.389	0.429	0.43
0.352	0.198	0.29	0.475
0.323	0.165	0.336	0.441
0.451	0.438	0.44	0.481
0.41	0.403	0.401	0.43
0.402	0.203	0.351	0.46
0.376	0.375	0.323	0.402
0.291	0.156	0.302	0.368
0.198	0.143	0.219	0.298
0.38	0.363	0.402	0.431
0.331	0.326	0.342	0.246

0.243	0.135	0.354	0.466
0.206	0.198	0.282	0.343
0.355	0.287	0.366	0.371
